# Understanding the associations between information sources, sociodemographics, and views on public health measures: evidence from the COVID-19 pandemic in Austria

**DOI:** 10.1186/s12889-024-19061-0

**Published:** 2024-06-12

**Authors:** Peter Gamillscheg, Susanne Mayer, Monika Pietrzak-Franger, Carina Hilmar, Alina Lange, Judit Simon, Agata Łaszewska

**Affiliations:** 1 Department of Health Economics, Center for Public Health, Medical University of Vienna, Kinderspitalgasse 15/1, Vienna, 1090 Austria; 2 Department of English and American Studies, University of Vienna, Spitalgasse 2, Hof 8.3 (Campus), Vienna, 1090 Austria; 3 Department of Psychiatry, University of Oxford, Warneford Hospital, Oxford, OX3 7JX UK

**Keywords:** Non-pharmaceutical intervention, Public health measure, Sociodemographic, COVID-19, Information source

## Abstract

**Background:**

Throughout the COVID-19 pandemic, it was a key priority for governments globally to ensure agreement with, and subsequently adherence to, imposed public health measures, specifically non-pharmaceutical interventions (NPIs). Prior research in this regard highlighted the role of COVID-19 information sources as well as sociodemographic and other personal characteristics, however, there is only limited evidence including both. To bridge this gap, this study investigated the associations of COVID-19 information sources such as social media and participant characteristics with agreement with and adherence to NPIs during the first lockdown in Austria.

**Methods:**

An online survey was conducted in May 2020 among adult Austrian residents asking about their experiences during the first lockdown. Collected data included sociodemographic characteristics, main COVID-19-related information sources, agreement with/adherence to three NPIs (no physical contact to family members not living in the same household, leisurely walks restricted to members of the same household, mandatory face masks) and information about perceived social support using the Multidimensional Scale of Perceived Social Support (MSPSS), anxiety/depression levels using the Hospital Anxiety and Depression Scale (HADS), whether participants felt well advised by the government, and whether participants perceived the pandemic to threaten their income. Ordered and multinomial logistic regression models were employed to achieve the research aims.

**Results:**

The cross-sectional sample consisted of 559 Austrian residents. Using social media as main COVID-19 information source was consistently associated with lower agreement with NPIs. A positive association with agreement with measures was found for higher educational backgrounds and higher anxiety levels. By contrast, higher levels of depression, not feeling well advised by the government, and perceiving the pandemic as an economic threat were negatively associated with agreement with measures. Moreover, the use of social media as main COVID-19 information source and not feeling well advised by the government were associated with lower adherence to NPIs. By contrast, higher levels of education were associated with higher adherence.

**Conclusions:**

This comprehensive analysis emphasizes the associations of COVID-19 information sources as well as sociodemographic and other participant characteristics with agreement with and adherence to NPIs, bearing important implications for future public health crisis communication strategies.

**Supplementary Information:**

The online version contains supplementary material available at 10.1186/s12889-024-19061-0.

## Background

In May 2023, the World Health Organization (WHO) declared COVID-19 as “*an established and ongoing health issue which no longer constitutes a public health emergency of international concern*” [1]. This announcement came more than three years after the initial declaration as such in January 2020 [2]. One of the major challenges policy makers were facing throughout the COVID-19 pandemic, and especially in its onset late 2019 and early 2020, was to ensure adherence to imposed public health measures, specifically non-pharmaceutical interventions (NPIs) such as wearing of face masks and social distancing [3]. At that time, media outlets took on an important role functioning as primary source of public health information, thereby connecting decision makers, experts, and the public [4–6].

In the early months of the COVID-19 pandemic, only limited answers could be provided to arising questions around the nature of the virus, potential cures, or what and how long the imposed NPIs would need to remain, as expertise was scarce and the situation rapidly evolved. This was intertwined with large-scale loss of life and wellbeing, especially among the most vulnerable population groups [7]. Lack of clear answers to these questions provided fertile ground for an *infodemic* raging in parallel to the pandemic. The infodemic was characterized by an information overload, both analogue and digital, with overwhelming, false or misleading information, thereby triggering confusion and unnecessary risk taking, as well as eroding trust in science, health authorities and imposed NPIs [8].

Previous research from Europe, including studies from Germany, Switzerland and Portugal, investigated media use practices among general populations during the COVID-19 pandemic [9–11]. Especially in the onset of the pandemic, the most commonly used information sources, trust in these sources, and channels used to share information were investigated. These studies found that the most frequent sources of information utilized during the pandemic were TV [9, 10] and digital media sources [9, 11]. Studies from the United States and South Korea moreover showed that the choice of COVID-19 information source was associated with sociodemographic factors [12, 13]. Among others, higher age and male gender were related to a lower usage of government websites and individuals with higher education were more likely to use CNN and less likely to use Fox News in the US analysis. While relying on doctors was associated with higher income in South Korea, its main association in the US was identification as ethnicity other than non-Hispanic White [12, 13].

In terms of the adherence to the imposed pandemic restrictions, international literature suggests that sociodemographic characteristics were associated with the perceived usefulness of and adherence to COVID-19-related NPIs. Relevant sociodemographic factors identified included employment status [14–16], education [16, 17] and related to this, health literacy [18, 19], age [16, 17, 20–24], gender [15–17, 20, 21, 23–25], family status [14, 15, 24], migration status [17] and ethnicity [21]. In addition, further associated participant characteristics included being part of a risk group, perceived mental and physical health, feeling well informed, and being economically affected by the pandemic [3]. Similarly, the relationship between preventative behaviors and information sources was also evident in literature [24, 26–30]. Yet, there is only limited research that considers both information sources and sociodemographic characteristics as predictors of agreement with and adherence to NPIs [31, 32]. Based on Canadian data, Courdi and colleagues found that using official sources and higher age were associated with higher adherence; however, they aggregated the collected information sources into official (including newspaper, TV, online news, and healthcare professionals as well as health authorities) and informal sources (including friends, family, and colleagues as well as people/groups on social media) and no differentiation between individual sources such as social media was included in their analyses [31]. Another study by Wu and Shen was based on Chinese data and identified higher adherence to be associated with the use of central government media, social media platform WeChat, higher age, female gender, and higher income while trust in the respective media amplified these effects. By contrast, using local media and social media platform Weibo were associated with lower adherence [32].

In line with other European countries, Austria entered its first lockdown on 15th March, 2020, effectively shutting down public life to a bare minimum in an effort to slow down the spread of the virus. The underlying regulation encompassed multiple specific restrictions such as mandatory face masks in public places, commuting to work only when necessary, closing all but essential shops and restricting physical contact to people living in the same household [33]. Previous research by Łaszewska et al. based on the same convenience sample used in this study showed that during the first lockdown about three-quarters of Austrian respondents agreed that the introduced NPIs were necessary [34]. At that time, restrictions on commuting and allowance of essential shopping only received the highest support. By contrast, contact restrictions and mandatory face masks received the least support among the studied respondents [34]. Irrespective of agreement, adherence to the imposed measures was high across the Austrians due to strong police enforcement through issuing fines [34, 35]. However, due to the sampling approach those results are not necessarily representative of the overall population [34]. Another study investigated predictors of adherent behavior during the pandemic in two Austrian states, identifying among others higher age and female gender to be associated with higher adherence [36]. However, neither study looked into information sources as potential predictor.

Considering the potential association of information sources with agreement/adherence and literature pointing to an association of individual sociodemographic and other participant characteristics with information sources [12, 13, 37, 38], this study focuses on comprehensively investigating the role of COVID-19 information sources such as social media during the first lockdown in Austria. Therefore, the specific aims of the study are to (i) analyze the association of sociodemographic and other participant characteristics with individual COVID-19 information sources; (ii) analyze the association of sociodemographic and other participant characteristics as well as individual COVID-19 information sources with agreement with imposed NPIs; and (iii) analyze the association of sociodemographic and other participant characteristics as well as individual COVID-19 information sources with adherence to imposed NPIs. Against the background of the presented literature, we expect to find participant characteristics to be associated with the use of different COVID-19 information sources. Moreover, we expect those participant characteristics as well as COVID-19 information sources to be associated with agreement with/adherence to NPIs. To the best of our knowledge, this has not yet been researched, neither in a European context nor at this level of detail.

## Methods

### Study sample and recruitment

The analyzed survey data refer to the first Austrian lockdown from March 15th to April 15th, 2020, and was collected from May 27th to June 16th, 2020. The study was conducted online following a convenience sampling approach, which means everybody with access to the survey and fulfilling the inclusion criteria could participate. Inclusion criteria were: at least 18 years of age, sufficient German skills to participate, and main residency in Austria at the time of the lockdown. The survey was conducted in German. Information material on the study, including a weblink to the survey, was distributed via social media and emails targeting a wide range of individuals and organizations across Austria such as universities, local governments, and non-profit organizations. Moreover, in an effort to achieve a diverse sample, the study information was posted in the Facebook comment sections of articles concerning COVID-19 shared by large Austrian newspapers spanning high quality to tabloid. Further details about the study design, survey methods and recruitment are available elsewhere [39], the data set is provided on Zenodo [40]. In total, the sample consisted of 560 participants. Conducted analyses were restricted to female and male gender identifications due to the small number of participants identifying as diverse (*n* = 1), resulting in a final sample size of 559.

### Analyzed variables

The data collected included sociodemographic and other participant characteristics, COVID-19 information sources, and information on agreement with and adherence to selected imposed NPIs.

#### Sociodemographic characteristics

Sociodemographic characteristics included age, gender, education, and migration background. Age was used as continuous and gender as binary variable in the analyses. Highest level of completed education answer options included “Primary school”, “Apprenticeship with vocational school”, “Technical or commercial school”, “A-levels”, “Any other higher degree following A-levels”, and “Degree from a university, (technical) college”. The variable was recoded to “Lower than A-levels”, “A-levels” (including non-university degrees), and “University degree“. Migration background answer options included “No migration background”, “EU before 2004/European Economic Area/Switzerland”, “EU after 2004”, “Former Yugoslavia (non-EU)/Turkey”, and “Other countries”. The variable was recoded into binary, combining all options other than “No migration background” versus “Migration background”.

#### Other participant characteristics

In addition, the following other self-reported variables that were identified as significant predictors of adherence to NPIs in previous research [3, 41–43] were included in the analyses: (i) mental health status; (ii) level of social support; (iii) having a chronic physical disease; (iv) perception of governmental communication; and (v) perception of an economic threat by the pandemic.

The mental health status was measured by assessing the general levels of anxiety and depression using the Hospital Anxiety and Depression Scale (HADS) instrument [44]. The instrument uses two sub-scales from 0 to 21 for anxiety and depression respectively, with a higher score indicating a higher level of anxiety or depression. The level of perceived social support was assessed using the Multidimensional Scale of Perceived Social Support (MSPSS) [45] where a higher score reflects better social support on a 0 to 7 scale. More details about the standardized instruments used in the survey are described elsewhere [39]. Self-reported information on chronic diseases (answer options: “Cancer”, “Cardiovascular diseases”, “Lung diseases such as asthma, cystic fibrosis, or COPD”, ”Liver diseases such as hepatitis”, “Stroke/cerebrovascular diseases”, “Diabetes”) was collected and coded into a binary variable representing at least one chronic disease versus none. The perception of governmental communication was assessed using a question asking participants whether they felt well advised by the government during the first lockdown (answer options: “Yes”, “No”). The perception of an economic threat was assessed by asking individuals whether the COVID-19 pandemic posed a threat to their income based on a five-point Likert scale (from “Strongly disagree” to “Strongly agree”). The variable was recoded to binary, combining “Strongly disagree”, “Disagree”, and “Neutral” to “No income threat or neutral” as well as “Strongly agree” and “Agree” to “Income threat”.

#### COVID-19 information sources

Participants chose their main COVID-19-related information source from a list including TV, newspaper, news websites, social media, government websites, international organization websites such as the WHO’s, and other sources. Participants had to select one single main information source. Family, friends, and colleagues as well as health professionals were not included as COVID-19 information sources in this study.

#### Non-pharmaceutical interventions (NPIs)

With respect to the NPIs designed to contain the spread of the virus, participants provided information in two separate, consecutive questions on the extent to which they agreed with and adhered to selected imposed measures. A 10-point scale defined as ordinal where 1 indicated no and 10 full agreement/adherence was used for assessment of the following three measures:

- No physical contact to family members not living in the same household.
- Leisurely walks only alone or with members of the shared household.
- Mandatory face masks on public transport and in opened stores.

Additionally, participants also indicated on a five-point Likert scale from “Strongly disagree” to “Strongly agree” to what extent they overall agreed with the NPIs in general.

### Data analysis

Descriptive sample statistics including frequencies and percentages were calculated and presented in comparison to the general Austrian population data. As the study aimed to investigate associations between sociodemographic and other characteristics, COVID-19 information sources, and agreement with and adherence to NPIs, three sets of regression models were employed:

- First, the association of sociodemographic as well as other participant characteristics with COVID-19 information sources was estimated with a multinomial logistic regression model, reflecting the categorical outcome data. Results were reported as relative risk ratios (RRR) and 95% confidence intervals (CI).
- Second, the association of COVID-19 information sources, sociodemographic and other participant characteristics with agreement with NPIs was estimated through ordered logistic regression models, reflecting the ordinal outcome data. Results were reported as odds ratios (OR) and 95% CIs.
- Third, the association of COVID-19 information sources, sociodemographic and other participant characteristics with adherence to NPIs was estimated using ordered logistic regression reported as ORs and 95% CIs.

Participants had the option not to answer individual questions. No imputation was conducted for missing values. Consequently, samples differed between regressions, as all participants who provided complete data for variables incorporated in the model were included.

The mean variance inflation factor (VIF) was calculated to test for multicollinearity and was 1.39 for the independent variables. While the majority (80%) of individual values were between 1.04 and 1.24, the HADS anxiety and depression sub-scores had a VIF of 2.47 and 2.58, respectively. Considering the research aims of this study and the known associations between sociodemographic characteristics and the outcome measures in question [46, 47], those VIFs pose no challenge to the validity of the analyses and multicollinearity was not of concern. Detailed VIF results for each variable can be found in Table A1 in the Supplementary material.

All analyses were conducted in Stata 17, considering a p-value of ≤ 0.05 as statistically significant. Given the exploratory nature of the study, no corrections for multiple hypothesis testing were applied.

## Results

### Sample characteristics

The detailed sample characteristics are presented in Table 1. 74% (*n* = 416) of the participants were women, 38% (*n* = 215) Viennese, 54% (*n* = 301) had a university degree. This compares to around 51% [48], 22% [49], and 20% [50], respectively, in the general Austrian population. The mean age was 40.2 (standard deviation 11.6). Observed participant characteristics suggest that the representativeness of the study sample of the overall population may be limited.

**Table 1:** Sociodemographic background of study participants (*n* = 559)

	Frequency	Percent	Austria (latest available year)
**Gender**			
Male	143	26%	49% (2023)
Female	416	74%	51% (2023)
**Total**	**559**	**100%**	Source: [48]
**Age**			
18–29	97	17%	18% (2023)
30–49	318	57%	35% (2023)
50–64	124	22%	29% (2023)
65–79	13	2%	18% (2023)
*Missing*	*7*	*1%*	
**Total**	**559**	**100%**	Source: [48]
**Region**			
Burgenland	12	2%	3% (2023)
Carinthia	41	7%	6% (2023)
Lower Austria	109	20%	19% (2023)
Upper Austria	66	12%	17% (2023)
Salzburg	25	4.%	6% (2023)
Styria	62	11%	14% (2023)
Tyrol	24	4%	9% (2023)
Vorarlberg	5	1%	4% (2023)
Vienna	215	38%	22% (2023)
**Total**	**559**	**100%**	Source: [49]
**Migration**			
No migration background	488	87%	74% (2022)
Migration background	66	12%	26% (2022)
*Missing*	*5*	*1%*	
**Total**	**559**	**100%**	Source: [59]
**Education**			
Lower than A-levels	126	22%	50% (2021)
A-levels	132	24%	30% (2021)
University	301	54%	20% (2021)
**Total**	**559**	**100%**	Source: [50]

Table 2 shows the distribution of main COVID-19 information sources with TV being the most popular choice at 37% (*n* = 205) followed by news websites at 27% (*n* = 153) and social media at 10% (*n* = 58). TV was the preferred information source for all presented sociodemographic subgroups except those participants with migration background who preferred news websites. The use of social media was more frequent among those with education lower than A-levels (17%), migration background (14%), and those not feeling well advised by the government (14%).

**Table 2:** Main COVID-19 information source during the first lockdown by sociodemographic characteristics

Main information source	*n*	%	Female (%)	Male (%)	Migration background (%)		No migration background (%)	Lower than A-levels (%)		A-levels (%)		University (%)	
			*n* = 416	*n* = 143	*n* = 66		*n* = 488	*n* = 126		*n* = 132		*n* = 301	
TV	205	37%	38%	34%	18%		39%	48%		36%		33%	
Newspaper	37	7%	8%	4%	3%		7%	3%		6%		8%	
News websites	153	27%	25%	34%	42%		26%	11%		32%		32%	
Government websites	46	8%	8%	8%	8%		8%	10%		7%		8%	
Social media	58	10%	11%	9%	14%		10%	17%		13%		7%	
International guidelines (e.g., WHO)	21	4%	4%	3%	5%		4%	2%		2%		5%	
Other	20	4%	3%	5%	5%		3%	2%		2%		5%	
I do not want to answer	6	1%	1%	2%	2%		1%	4%		-		0%	
*Missing*	13	2%	2%	3%	5%		2%	4%		2%		2%	
**Total**	**559**	**100%**	**100%**	**100%**	**100%**		**100%**	**100%**		**100%**		**100%**	
***Main information source***	***n***	***%***	***Feeling well advised by the government *** ***(%)***			***Not feeling well advised by the government (%)***			***No income threat (%)***		***Income threat (%)***		
			**n = 370**			**n = 172**			**n = 390**		**n = 165**		
TV	205	37%	42%			25%			40%		29%		
Newspaper	37	7%	7%			6%			7%		5%		
News websites	153	27%	26%			29%			25%		32%		
Government websites	46	8%	8%			8%			8%		8%		
Social media	58	10%	9%			14%			9%		13%		
International guidelines (e.g., WHO)	21	4%	3%			5%			3%		5%		
Other	20	4%	3%			5%			4%		2%		
I do not want to answer	6	1%	0%			3%			1%		2%		
*Missing*	13	2%	2%			4%			2%		2%		
**Total**	**559**	**100%**	**100%**			**100%**			**100%**		**100%**		

**Fig. 1:**
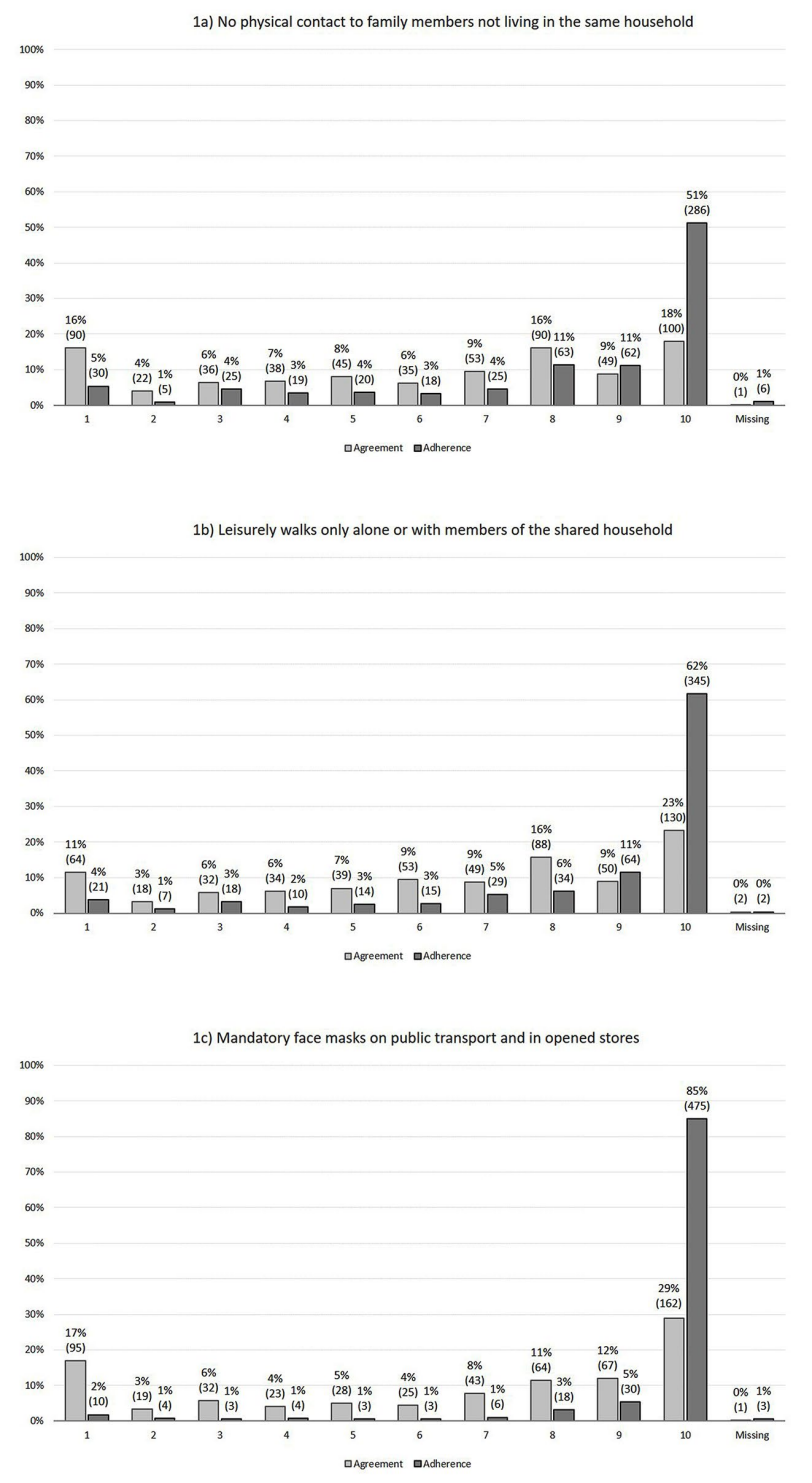
Response distribution agreement with and adherence to three NPIs in % and total

Figure 1 presents the distribution of answers regarding agreement with and adherence to imposed NPIs. For all three NPIs, the highest share of participants fully agreed/adhered. However, the share of highest agreement with and highest adherence to an NPI differed vastly, with more participants fully adhering than fully agreeing. The largest difference in this regard was observed for mandatory face masks with 29% fully agreeing compared to 85% fully adhering. By contrast, 17% of participants did not agree with this NPI at all but only 2% did not adhere at all.

#### Association of sociodemographic and other participant characteristics with COVID-19 information sources

Regression analysis showed older participants were more likely to use TV (base case) compared to social media (RRR 0.97, 95% CI 0.94, 1.00) (Table 3). Participants with migration background were found to prefer news websites (RRR 2.91, 95% CI 1.28, 6.62) and social media (RRR 4.14, 95% CI 1.57, 10.93) compared to TV. Participants with a university degree mainly used newspaper (RRR 4.44, 95% CI 1.20, 16.37) and news websites (RRR 4.31, 95% CI 2.08, 8.93) and less often social media (RRR 0.42, 95% CI 0.19, 0.96) as a preferred information source over TV, compared to those with education lower than A-levels. This preference of newspaper and news websites over TV by those with a university degree also showed the largest relative risk ratios of this analysis at 4.44 and 4.31, respectively. By contrast, participants with A-levels as highest level of education were only found to prefer news websites over TV compared to those with a below-A-levels education (RRR 3.19, 95% CI 1.42, 7.16).

**Table 3:** Association of sociodemographic and other characteristics with COVID-19 information sources

Base case: TV	Newspaper	News websites	Government websites	Social media
*N* = 497	*Relative risk ratio [95% CI]*	*Relative risk ratio [95% CI]*	*Relative risk ratio [95% CI]*	*Relative risk ratio [95% CI]*
Age	1.020 [0.985,1.057]	0.978 [0.955,1.001]	0.973 [0.945,1.002]	0.966* [0.938,0.996]
Female gender	1.582 [0.559,4.476]	0.677 [0.386,1.188]	0.932 [0.430,2.023]	1.217 [0.572,2.590]
Migration background	0.873 [0.203,3.762]	2.905* [1.275,6.619]	1.659 [0.483,5.695]	4.141** [1.569,10.931]
*Education level lower than A-levels (base case)*				
A-levels	3.175 [0.736,13.693]	3.191** [1.422,7.163]	0.855 [0.317,2.304]	0.601 [0.242,1.488]
University	4.440* [1.204,16.367]	4.310*** [2.081,8.926]	1.328 [0.559,3.158]	0.421* [0.185,0.960]
Not feeling well advised by the government	1.810 [0.812,4.033]	1.549 [0.894,2.683]	1.856 [0.877,3.928]	2.707** [1.332,5.503]
HADS anxiety sub-score	1.220** [1.069,1.393]	1.080 [0.987,1.182]	1.065 [0.928,1.222]	0.907 [0.797,1.032]
HADS depression sub-score	0.774** [0.639,0.937]	0.944 [0.858,1.040]	0.863* [0.751,0.991]	1.132 [0.984,1.301]
Total MSPSS score	1.148 [0.792,1.663]	0.778* [0.620,0.975]	0.872 [0.637,1.075]	0.995 [0.742,1.335]
Chronic disease	0.392 [0.085,1.805]	0.368* [0.160,0.846]	1.332 [0.536,3.310]	0.217 [0.044,1.062]
Perceived income threat as a result of the pandemic	1.008 [0.388,2.618]	1.616 [0.924,2.826]	1.203 [0.540,2.678]	1.691 [0.844,3.390]

HADS = Hospital Anxiety and Depression Scale, MSPSS = Multidimensional Scale of Perceived Social Support.

* p-value = < 0.05, ** p-value = < 0.01, *** p-value = < 0.001

Those participants not feeling well advised by the government preferred social media (RRR 2.71, 95% CI 1.33, 5.50) over TV as main COIVD-19 information source. Participants with higher depression levels chose TV over government websites (RRR 0.86, 95% CI 0.75, 0.99) and newspaper (RRR 0.77, 95% CI 0.64, 0.94). By contrast, those with higher anxiety levels preferred newspaper to TV (RRR 1.22, 95% CI 1.07, 1.39). Moreover, those with either higher perceived social support (RRR 0.78, 95% CI 0.62, 0.98) or a chronic disease (RRR 0.37, 95% CI 0.16, 0.85) preferred TV compared to news websites. The latter also constituted the lowest relative risk ratio at 0.37 (95% CI 0.16, 0.85), in other words the strongest preference of TV over another information source.

In terms of robustness, all presented associations of sociodemographic and other participant characteristics with COVID-19 information sources showed a clear direction considering the respective relative risk ratios and 95% confidence intervals besides the association of age and social media usage over TV, which included an upper bound of 1.00 (rounded from 0.996, see Table 3). The categories of COVID-19 information sources “International guidelines” and “Other” were not presented in Table 3 due to the negligible number of participants reporting the use of these sources in our sample.

#### Association of COVID-19 information sources, sociodemographic and other participant characteristics with agreement with NPIs

Looking at COVID-19 information sources, regression analyses showed that using social media as main COVID-19 information source was consistently associated with lower agreement with all individual NPIs (no contact: OR 0.47, 95% CI 0.24, 0.93; leisurely walks: OR 0.49, 95% CI 0.27, 0.88; mandatory masks: OR 0.45, 95% CI 0.23, 0.89) and overall agreement (OR 0.44, 95% CI 0.21, 0.91) with the measures compared to those preferring TV (Table 4). Furthermore, those using news websites rather than TV were found to display lower overall agreement (OR 0.62, 95% CI 0.39, 0.97) as well as lower agreement with restricted leisurely walks (OR 0.63, 95% CI 0.41, 0.96).

**Table 4:** Association of sociodemographic and other characteristics and COVID-19 information sources with agreement with NPIs

Level of agreement	No contact	Leisurely walks	Mandatory masks	Overall agreement
*n* = 496	*Odds ratio [95% CI]*	*Odds ratio [95% CI]*	*Odds ratio [95% CI]*	*Odds ratio [95% CI]*
*TV-news (base case)*				
Newspapers	0.886 [0.425,1.845]	0.646 [0.308,1.358]	2.026 [0.891,4.609]	0.714 [0.341,1.491]
News websites	0.976 [0.653,1.458]	0.628* [0.412,0.957]	0.740 [0.488,1.123]	0.616* [0.393,0.966]
Government websites	0.913 [0.519,1.607]	0.998 [0.565,1.763]	1.240 [0.693,2.220]	1.173 [0.587,2.341]
Social media	0.471* [0.237,0.934]	0.485* [0.267,0.883]	0.452* [0.230,0.888]	0.435* [0.209,0.906]
International guidelines (e.g. WHO)	0.912 [0.398,2.093]	1.080 [0.432,2.701]	1.269 [0.487,3.311]	0.637 [0.230,1.761]
Other sources	1.254 [0.629,2.503]	1.135 [0.385,3.346]	0.829 [0.403,1.709]	0.722 [0.278,1.875]
Age	0.990 [0.976,1.005]	1.006 [0.991,1.022]	1.002 [0.987,1.018]	0.989 [0.973,1.006]
Female gender	0.761 [0.540,1.075]	1.019 [0.693,1.498]	1.098 [0.758,1.592]	0.974 [0.623,1.523]
Migration background	1.052 [0.595,1.861]	1.100 [0.629,1.925]	2.121** [1.243,3.619]	0.961 [0.530,1.742]
*Education level lower than A-levels (Base case)*				
A-levels	1.654 [0.950,2.878]	1.355 [0.822,2.234]	2.784*** [1.643,4.717]	1.832* [1.034,3.247]
University	1.726* [1.088,2.738]	1.280 [0.825,1.984]	2.424*** [1.574,3.735]	2.213*** [1.387,3.530]
Not feeling well advised by the government	0.198*** [0.137,0.286]	0.233*** [0.158,0.344]	0.220*** [0.147,0.327]	0.168*** [0.113,0.251]
HADS anxiety sub-score	1.083* [1.019,1.151]	1.120*** [1.049,1.195]	1.071* [1.004,1.142]	1.147*** [1.069,1.230]
HADS depression sub-score	0.904** [0.839,0.974]	0.908** [0.847,0.973]	0.926* [0.865,0.993]	0.869*** [0.806,0.937]
Total MSPSS score	1.078 [0.914,1.271]	1.097 [0.942,1.278]	1.017 [0.874,1.184]	1.115 [0.947,1.313]
Chronic disease	1.166 [0.705,1.928]	1.179 [0.636,2.186]	1.706 [0.982,2.964]	1.860* [1.037,3.336]
Perceived income threat as a result of the pandemic	0.590** [0.404,0.863]	0.836 [0.559,1.250]	0.782 [0.539,1.134]	0.632* [0.421,0.947]

HADS = Hospital Anxiety and Depression Scale, MSPSS = Multidimensional Scale of Perceived Social Support, level of adherence measured on ordinal scales

* p-value ≤ 0.05, ** p-value ≤ 0.01, *** p-value ≤ 0.001

Regarding participant characteristics, having a university degree was consistently associated with higher agreement with individual imposed NPIs (no contact: OR 1.73, 95% CI 1.09, 2.74; mandatory masks: OR 2.42, 95% CI 1.57, 3.74) and overall agreement (OR 2.21, 95% CI 1.39, 3.53) compared to those with an education lower than A-levels (Table 4). Further highlighting the role of education, also those with A-levels were found to show higher agreement than those without regarding mandatory masks (OR 2.78, 95% CI 1.64, 4.72) and overall (OR 1.83, 95% CI 1.03, 3.25). Migration background was associated with higher support for mask wearing (OR 2.12, 95% CI 1.24, 3.62). Moreover, not feeling well advised by the government (no contact: OR 0.20, 95% CI 0.14, 0.29; leisurely walks: OR 0.23, 95% CI 0.16, 0.34; mandatory masks: OR 0.22, 95% CI 0.15, 0.33; overall: OR 0.17, 95% CI 0.11, 0.25) and higher depression levels (no contact: OR 0.90, 95% CI 0.84, 0.97; leisurely walks: OR 0.91, 95% CI 0.85, 0.97; mandatory masks: OR 0.93, 95% CI 0.87, 0.99; overall: OR 0.87, 95% CI 0.81, 0.94) were consistently associated with lower agreement with all individual NPIs and overall. By contrast, higher anxiety levels (no contact: OR 1.08, 95% CI 1.02, 1.15; leisurely walks: OR 1.12, 95% CI 1.05, 1.20; mandatory masks: OR 1.07, 95% CI 1.00, 1.14; overall: OR 1.15, 95% CI 1.07, 1.23) increased the agreement with NPIs as did having a chronic disease for overall agreement (OR 1.86, 95% CI 1.04, 3.34). Moreover, a perceived income threat was associated with lower overall agreement (OR 0.63, 95% CI 0.42, 0.95) and lower agreement with restrictions on physical contact (OR 0.59, 95% CI 0.40, 0.86).

The lowest odds ratios were found for not feeling well advised by the government between 0.17 (95% CI 0.11, 0.25) and 0.23 (95% CI 0.16, 0.34) for agreement overall and with restricted leisurely walks respectively. This compares with the highest odds ratios found for those with A-levels as highest completed education at between 1.83 (95% CI 1.03, 3.25) and 2.78 (95% CI 1.64, 4.72) for overall agreement and agreement with mandatory masks respectively. All presented associations of COVID-19 information sources, sociodemographic and other participant characteristics with agreement with NPIs showed a clear direction considering the respective odds ratios and 95% confidence intervals besides the association of anxiety levels and agreement with mandatory masks, which included a lower bound of 1.00 (rounded from 1.004, see Table 4). However, the associations of anxiety levels and agreement with the individual NPIs were consistently significant.

#### Association of COVID-19 information sources, sociodemographic and other participant characteristics with adherence to NPIs

In terms of the main COVID-19 information sources, regression analyses showed that only social media (OR 0.41, 95% CI 0.17, 1.00) was statistically significantly associated with lower adherence to mandatory masks compared to those preferring TV (Table 5). Furthermore, female gender (OR 2.44, 95% CI 1.35, 4.38) was positively associated with higher adherence to mandatory masks as was having A-levels as highest completed education compared to those without A-levels (OR 2.42, 95% CI 1.00, 5.84). The latter also was associated with higher adherence to restrictions on leisurely walks (OR 2.16, 95% CI 1.22, 3.82) as was having a university degree (OR 2.39, 95% CI 1.47, 3.88). Moreover, having a chronic disease was associated with higher adherence to personal contact restrictions (OR 1.87, 95% CI 1.07, 3.26). Higher anxiety levels were positively associated with adherence to personal contact NPIs (OR 1.09, 95% CI 1.02, 1.17). Not feeling well advised by the government was the only variable found to have statistically significant associations with all three NPIs and consistently reduced adherence (no contact: OR 0.43, 95% CI 0.29, 0.65; leisurely walks: OR 0.31, 95% CI 0.20, 0.48; mandatory masks: OR 0.31, 95% CI 0.17, 0.57). Overall, fewer associations were identified for adherence with NPIs than for agreement with them.

**Table 5:** Association of sociodemographic and other characteristics and COVID-19 information sources with adherence to NPIs

Level of adherence	*No contact*(*n* = 493)	*Leisurely walks *(*n* = 496)	*Mandatory masks *(*n* = 495)
	*Odds ratio [95% CI]*	*Odds ratio [95% CI]*	*Odds ratio [95% CI]*
*TV-news (base case)*			
Newspapers	0.564 [0.280,1.136]	1.806 [0.707,4.612]	4.942 [0.555,43.982]
News websites	1.150 [0.728,1.817]	0.826 [0.510,1.340]	0.842 [0.424,1.673]
Government websites	1.125 [0.630,2.008]	1.330 [0.670,2.642]	1.888 [0.619,5.755]
Social media	0.700 [0.368,1.332]	0.638 [0.326,1.247]	0.413* [0.171,1.000]
International guidelines, e.g. WHO	1.412 [0.524,3.803]	1.099 [0.348,3.468]	3.736 [0.373,37.417]
Other sources	2.850 [0.966,8.409]	1.032 [0.311,3.419]	1.900 [0.300,12.055]
Age	0.995 [0.978,1.011]	1.010 [0.992,1.029]	0.994 [0.972,1.017]
Female gender	1.147 [0.760,1.731]	1.103 [0.695,1.751]	2.435** [1.354,4.378]
Migration background	1.194 [0.595,2.394]	1.064 [0.495,2.291]	0.842 [0.355,1.996]
*Education level lower than A-levels (base case)*			
A-levels	1.324 [0.770,2.275]	2.157** [1.219,3.817]	2.419* [1.002,5.839]
University	1.240 [0.774,1.986]	2.385*** [1.467,3.878]	1.609 [0.841,3.081]
Not feeling well advised by the government	0.431*** [0.286,0.647]	0.305*** [0.195,0.476]	0.306*** [0.165,0.568]
HADS anxiety sub-score	1.091* [1.018,1.169]	1.071 [0.997,1.151]	1.055 [0.941,1.183]
HADS depression sub-score	0.947 [0.877,1.021]	0.987 [0.912,1.069]	1.012 [0.901,1.137]
Total MSPSS score	0.980 [0.827,1.161]	1.109 [0.935,1.314]	1.108 [0.843,1.457]
Chronic disease	1.869* [1.070,3.264]	1.613 [0.862,3.017]	0.888 [0.366,2.159]
Perceived income threat as a result of the pandemic	0.695 [0.474,1.021]	0.889 [0.573,1.381]	0.884 [0.482,1.618]

*Note* HADS = Hospital Anxiety and Depression Scale, MSPSS = Multidimensional Scale of Perceived Social Support, level of adherence measured on ordinal scales

* p-value ≤ 0.05, ** p-value ≤ 0.01, *** p-value ≤ 0.001

Similar to the analysis on agreement with NPIs, not feeling well advised by the government showed the lowest odds ratios at 0.31 for adherence to restrictions on leisurely walks and to mandatory masks. Moreover, A-levels or a university degree as highest level of education again showed high odds ratios at between 2.16 (95% CI 1.22, 3.82) and 2.42 (95% CI 1.00, 5.84) for adherence to restrictions on leisurely walks and to mandatory masks, only trumped by female gender with an odds ratio of 2.44 (95% CI 1.35, 4.38) for adherence to mandatory masks. All presented associations of COVID-19 information sources, sociodemographic and other participant characteristics with adherence to NPIs showed a clear direction considering the respective odds ratios and 95% confidence intervals besides the associations of social media and A-levels as highest level of education with adherence to mandatory masks, which included an upper (rounded from 0.9995) and lower bound (rounded from 1.002, see Table 5) of 1.00, respectively.

## Discussion

### Main findings

In this study, we showed the association - and importance - of the choice of COVID-19 information source, particularly social media, and sociodemographic and other characteristics, particularly education, with agreement with imposed NPIs during the first lockdown in Austria. Moreover, we found that feeling well advised by the government as well as anxiety and depression levels were significantly associated with agreement with NPIs, with the variable reflecting government advice consistently showing the largest magnitude of effect sizes, corresponding to lower agreement by those not feeling well advised. Our study pointed out that adherence, by contrast, did not differ much across the population subgroups, which might reflect strict legal consequences in case of non-adherence, resulting in population-wide high adherence to all imposed measures. Not feeling well advised by the government was the only exception, which was consistently associated with lower adherence across NPIs and A-levels or a university degree by contrast with higher adherence to individual NPIs compared to those with an education level below A-levels. COVID-19 information sources, however, did not show any associations with adherence besides social media being associated with lower adherence to mandatory masks compared to the base group preferring TV.

Looking into the COVID-19 information source selection, we identified age, migration background, education, depression, perceived social support, chronic diseases, and not feeling well advised by the government to be associated. Participants of older age and those with chronic diseases, higher depression levels, or higher perceived social support relied on TV, while A-levels or a university degree were associated with information sources such as newspapers and news websites rather than TV. Migration background in turn was associated with higher use of news websites and social media.

#### What we know already

In our study, with a high demand for the most up to date information potentially related to the dynamically evolving situation, 37% referred to TV as their main COVID-19-related information source followed by news websites (27%) and social media (10%). While this ranking is in line with the Reuters Digital News Report, it misses newspapers that were only named by 7% of the sample as main information source despite Austrians’ heavy reliance on this medium with 40% of Austrians still accessing newspapers at least once a week in 2023 (51% in 2020 at the time of data collection) [51, 52].

The Austrian Corona Panel Project looked into the demand for COVID-19-related information in April 2020. Their data showed that the majority of participants frequently utilized both traditional (80%) and social (57%) media daily which partly confirmed results of this study [53]. Other Austrian studies highlighted that the level of trust in media reports on COVID-19 as well as the level of misinformation differed by accessed information source [54, 55]. Those accessing alternative media *“representing views different to societal consensus”* or social media more than once per week showed lower trust in COVID-19 media reports in general [54]. Furthermore, only 40% of participants managed to identify false claims and 12% could not debunk any at all [55].

At the same time, there were notable sociodemographic differences in the audience of the respective information sources. While the largest Austrian tabloid “Kronen Zeitung” and commercial TV were frequented more often by participants with lower education levels, those with higher education and higher income tended towards higher quality newspapers “Die Presse” and “Der Standard” [56]. While we did not differentiate between different qualities of information sources or individual media outlets, these findings are in line with our study, as we also point out the sociodemographic differences in used COVID-19 information sources. However, in contrast to our results, the Austrian Corona Panel Project did not find any significant differences in social media and TV usage by education [56].

### What this study contributes

In line with international literature [12, 21, 25, 31, 37], we identified associations between sociodemographic and other participant characteristics, COVID-19 information sources, and agreement with and adherence to NPIs in Austria, for which evidence was lacking thus far. We moreover contribute with a comprehensive Austrian perspective to partially comparable international studies, first and foremost Margraf and colleagues [3], covering France, Germany, Poland, Russia, Spain, Sweden, UK, and the US. We confirmed their findings especially regarding the association of a perceived economic threat, mental health, not feeling well informed, and having a chronic disease with agreement with NPIs. However, we did not find conclusive evidence on the role of female gender and a higher age. Yet comparability is limited, as Margraf et al. [3] did not include COVID-19 information sources in their analysis as we did. Studies including both COVID-19 information sources and sociodemographic characteristics were conducted in Canada and China [31, 32]. However, the Canadian analysis did not allow for the detailed comparison between information sources as described in the current study because it only differentiated between official and informal sources [31]. Moreover, findings are difficult to put into Austrian context because of the peculiarities of the Austrian population when it comes to media use. Examples are the high reliance on TV for news, with 68% of the Austrian population regularly using the medium compared to 60% in Canada in 2020 (59% and 49% in 2023), and on print media, with 51% in Austria compared to 25% in Canada in 2020 (40% and 14% in 2023) [51, 52]. A comparison with China, on the other hand, may be considered problematic given the high levels of content censorship. China placed 177th out of 180 countries in the 2020 Reporters without Borders Press freedom index compared to Austria, which ranked 18th [57, 58].

Our comprehensive analysis of associates of agreement with/adherence to NPIs particularly highlights the role of social media in shaping people’s opinions about COVID-19-related measures as it was consistently associated with lower agreement. This could be due to different narratives provided by different information sources as well as varying amounts of unmoderated misleading or false content and can be of a high policy relevance.

This study furthermore identified associates of COVID-19 information sources, thereby contributing to future research on the potential role of information sources as mediator or moderator in the association of sociodemographic and other characteristics with agreement with/adherence to NPIs.

#### Limitations

Generalization of our findings is limited due to the sample size and composition. The sample analyzed in this analysis was selective as a result of the convenience sampling recruitment and contained more women and a higher share of university degrees than the Austrian population. Statistical uncertainties and the risk of type-2 errors in the presented associations should be considered when interpreting the results as there were small group sizes for some subgroups in the analysis and some results – while statistically significant – included 1.00 as one of the 95% confidence interval bounds. However, we consider our main findings robust, as they are statistically significant across multiple NPIs. This is the case for social media or news website preference, A-levels or university degree, not feeling well advised by the government, and HADS anxiety and depression scores as well as a perceived income threat for agreement with NPIs. For adherence to NPIs, only A-levels and not feeling well advised by the government were statistically significant for more than one NPI. Other reported individual findings could potentially be the result of the abovementioned statistical uncertainties.

Moreover, the survey was conducted in German and online only, hence a potential selection bias might affect results due to language and digital literacy barriers. As the data were collected in retrospect after the lockdown ended, other potential biases include recall/response or confirmation bias. Also, only highly aggregated information sources such as TV, newspaper, or social media were considered as choices in the survey, but no detailed options such as different TV channels or specific social media such as Facebook, Instagram or TikTok were included. Hence, it was not possible to gain a deeper understanding of the specific narrative and quality of information participants were exposed to. Correction for multiple testing was not applied in this study, yet we consider the results to be robust due to their consistency across different regression models. Lastly, as our data are cross-sectional, we cannot draw conclusions about the flow of causality between the observed variables.

## Conclusion

Our findings emphasize the associations of sociodemographic and other participant characteristics as well as COVID-19 information sources, in particular social media, with agreement with NPIs in Austria during an acute public health crisis. Future initiatives should take these results into account particularly in the design of communication strategies targeting population groups shown to display lower agreement with measures such as individuals with low education levels. This will allow to better engage with different sociodemographic groups via the respectively relevant information channels. Our results highlight starting points for important future research: longitudinal data may complement the findings of the presented study through the identification of causal effects, while evaluating the impact of information sources on agreement with/adherence to NPIs on a more detailed level, including different newspapers, TV programs, or social media platforms/content creators.

### Electronic supplementary material

Below is the link to the electronic supplementary material.

Supplementary Material 1

## Data Availability

The questionnaire is available as a supplementary material to a previous publication by Laszewska et al. 2021. The dataset analysed in the current study is available in the Zenodo repository, DOI 10.5281/zenodo.4598820 under https://zenodo.org/records/4598821.

## Abbreviations

CI: Confidence interval
HADS: Hospital Anxiety and Depression Scale
MSPSS: Multidimensional Scale of Perceived Social Support
NPI: Non-pharmaceutical intervention
OR: Odds ratio
RRR: Relative risk ratio
VIF: Variance inflation factor
WHO: World Health Organization
