# Supplementary material for: Understanding the associations between information sources, sociodemographics, and views on public health measures: evidence from the COVID-19 pandemic in Austria
Source: BMC Public Health. 2024 Jun 12;24:1576. doi: 10.1186/s12889-024-19061-0 (PMC11167807; doi:10.1186/s12889-024-19061-0)
Supplement: Supplementary file 1 — Supplementary Material 1 [file 12889_2024_19061_MOESM1_ESM.docx]

# ***Supplementary material***

# **Understanding the associations between information sources, sociodemographics, and views on public health measures: Evidence from the COVID-19 pandemic in Austria**

Peter Gamillscheg^1^, Susanne Mayer^1^*, Monika Pietrzak-Franger^2^, Carina Hilmar^2^, Alina Lange^2^, Judit Simon^1,3^, Agata Łaszewska^1^

^1^ *Medical University of Vienna, Department of Health Economics, Center for Public Health, Kinderspitalgasse 15/1, 1090 Vienna, Austria*

*^2^ University of Vienna, Department of English and American Studies, Spitalgasse 2, Hof 8.3 (Campus), 1090 Vienna, Austria*

*^3^ Department of Psychiatry, University of Oxford, Warneford Hospital, Oxford OX3 7JX, United Kingdom*

*Corresponding author: Susanne Mayer, Department of Health Economics, Center for Public Health, Medical University of Vienna, Kinderspitalgasse 15/1, 1090 Vienna, Austria, susanne.mayer@meduniwien.ac.at

**ORCID:**

Peter Gamillscheg: 0009-0002-4286-2285

Susanne Mayer: 0000-0002-4448-0478

Judit Simon: 0000-0001-9279-8627

Agata Laszewska: 0000-0002-6689-3708

Table A1. Multicollinearity diagnostics using the variance inflation factor (VIF)

| ***Extended model*** | ***VIF*** | ***Square root VIF*** | ***Tolerance*** | ***R-squared*** |
| --- | --- | --- | --- | --- |
| Age | 1.09 | 1.04 | 0.9172 | 0.0828 |
| Gender | 1.06 | 1.03 | 0.9391 | 0.0609 |
| Migration background | 1.06 | 1.03 | 0.9420 | 0.058 |
| Education | 1.04 | 1.02 | 0.9608 | 0.0392 |
| Feeling well advised by the government | 1.14 | 1.07 | 0.8738 | 0.1262 |
| HADS anxiety sub-scale | 2.47 | 1.57 | 0.4051 | 0.5949 |
| HADS depression sub-scale | 2.58 | 1.61 | 0.3882 | 0.6118 |
| MSPSS score | 1.24 | 1.11 | 0.8094 | 0.1906 |
| Having a chronic disease | 1.07 | 1.03 | 0.9376 | 0.0828 |
| Considering COVID-19 an income threat | 1.12 | 1.06 | 0.8908 | 0.0609 |
| **Mean** | **1.39** |  |  |  |
